# Human microvasculature-on-a chip: anti-neovasculogenic effect of nintedanib in vitro

**DOI:** 10.1007/s10456-018-9631-8

**Published:** 2018-07-02

**Authors:** Soheila Zeinali, Colette A. Bichsel, Nina Hobi, Manuela Funke, Thomas M. Marti, Ralph A. Schmid, Olivier T. Guenat, Thomas Geiser

**Affiliations:** 1 Organs-on-Chip Technologies Laboratory, ARTORG Center, University of Bern, Bern, Switzerland; 2 Vascular Biology Program, Harvard Medical School, Boston Children’s Hospital, Boston, MA USA; 3 Department of Pulmonary Medicine, Inselspital, Bern University Hospital, University of Bern, Bern, Switzerland; 4 Department for BioMedical Research, University of Bern, Bern, Switzerland; 5 Division of General Thoracic Surgery, Inselspital, Bern University Hospital, University of Bern, Bern, Switzerland

**Keywords:** Microvasculature-on-a chip, Nintedanib, Neovasculogenesis, Vascular permeability, Idiopathic pulmonary fibrosis

## Abstract

Idiopathic pulmonary fibrosis is characterized by a progressive scarring and stiffening of the peripheral lung tissue that decreases lung function. Over the course of the disease, the lung microvasculature undergoes extensive remodeling. There is increased angiogenesis around fibrotic foci and an absence of microvessels within the foci. To elucidate how the anti-fibrotic drug nintedanib acts on vascular remodeling, we used an in vitro model of perfusable microvessels made with primary endothelial cells and primary lung fibroblasts in a microfluidic chip. The microvasculature model allowed us to study the impact of nintedanib on permeability, vascularized area, and cell–cell interactions. The anti-vasculogenic impact of nintedanib was visible at the minimal concentrations of 10 nM, showing a significant increase in vessel permeability. Furthermore, nintedanib decreased microvessel density, diameter, and influenced fibroblast organization around endothelial microvessels. These results show that nintedanib acts on the endothelial network formation and endothelial–perivascular interactions. Advanced in vitro microvasculature models may thus serve to pinpoint the mechanistic effect of anti-fibrotic drugs on the microvascular remodeling in 3D and refine findings from animal studies.

**Electronic supplementary material:**

The online version of this article (10.1007/s10456-018-9631-8) contains supplementary material, which is available to authorized users.

## Introduction

Idiopathic pulmonary fibrosis (IPF) is a chronic and progressive pulmonary disorder, where progressive scarring of the peripheral lung ultimately leads to loss of lung function. IPF has a poor survival prognosis, a median survival of 2–3 years after diagnosis [1]. Some patients progress slowly, while others have episodes of acute exacerbations which lead to a quick decline in lung function [2]. The underlying cause of IPF is still unknown, but several risk factors have been identified. Smoking, exposure to pollutants, genetic predisposition, viral infection, gastro-esophageal reflux and aging increase the risk of developing IPF [1].

Typically, IPF patients have regions of stiff fibrotic tissue, which look like honeycombs on the macroscale. In these regions, extracellular matrix secreted by myofibroblasts accumulates, and there are dilated spaces, disruption of the alveolar barrier and epithelial hyperplasia [3]. IPF not only alters the normal lung parenchyma by progressive fibroblast proliferation and extensive deposition of extracellular matrix (ECM), but also non-homogenously remodels the pulmonary microvascular and macrovascular architecture [4]. Density of blood vessels in fibrotic regions is less, while adjacent non-fibrotic tissue is highly vascularized [5]. It is unclear whether neovascularization is a compensatory mechanism or whether it rather promotes disease progression.

Studies on IPF therapeutic compounds have largely focused on fibrosis and the knowledge is limited regarding IPF-associated vascular remodeling. However, recent findings suggest that the underlying pathobiology in IPF is much more intricate, and associates with a complex interaction between epithelial cells, fibroblasts, and endothelial cells [5]. These complex interactions are regulated by variety of angiogenesis promoters, inhibitors, and growth factors [5].

More light is starting to be shed on IPF pathogenesis with the introduction of tyrosine kinase inhibitors used to treat IPF. Nintedanib is a potent small molecule that inhibits the angiogenic receptor tyrosine kinases of fibroblast growth factor receptor (FGFR), vascular endothelial growth factor receptor (VEGFR), and platelet driven growth factor receptor (PDGFR) [6]. The in vitro efficacy of nintedanib was explored in vascular endothelial growth factor (VEGF)- or fibroblast growth factor (FGF)-2-stimulated human umbilical vascular endothelial cells (HUVECs), pericytes stimulated with platelet driven growth factor (PDGF) and FGF-2, and TGF-β-induced primary culture of lung fibroblasts from IPF patients [7]. Nintedanib induced apoptosis in HUVECs and pericytes in a concentration-dependent manner, and reduced collagen secretion and deposition by IPF fibroblasts [6]. The anti-fibrotic, anti-inflammatory, and anti-angiogenic effects of nintedanib were demonstrated in models of silica-induced lung fibrosis in mice and bleomycin-induced lung fibrosis in mice and rats [8, 9]. Recently, a bleomycin induced mouse model of lung fibrosis provided evidence that the anti-angiogenic activity of nintedanib recovered pulmonary microvasculature architecture and improved lung function in pulmonary fibrosis [10]. However, additional studies are needed to elucidate the mechanism of angiogenesis and vasculogenesis inhibition after treatment with nintedanib [10]. Differences in the cellular responses observed in standard in vitro studies, animal models, and human clinical studies may be confounding and hinder the efficient development of new therapeutic strategies. This is especially true for disease such as IPF which is known for complex correlation of fibrosis, angiogenesis and growth factor signaling [11]. Advanced in vitro microvasculature models that mimic in vivo conditions of human lung are one strategy to better understand the pulmonary vascular remodeling and investigate the efficacy of therapeutic compounds.

Recently, thanks to the development of advanced 3D in vitro models, the feasibility of self-assembled perfusable microvessel networks has been established [12–16]. Such models have several important features of the vascular microenvironment in vivo, and enable fine spatial and temporal resolution during in vitro experiments [14]. Based on an in vitro vasculogenesis model using co-culture of endothelial cells and pericytes, Bichsel et al. [12] described the biomimetic model of lung parenchyma microvasculature. This microfluidic co-culture system enabled the formation of a microvessel network that resembles human lung microvasculature in terms of morphology, vascular marker expression, permeability, and vasoactive response [12]. In another study, vessel formation by angiogenesis and vasculogenesis was achieved by co-culture of endothelial cells and human lung fibroblasts [13]. This vascular network displayed integral barrier function and long-term vascular stability [13]. These in vitro microfluidic models mimic the in vivo microenvironment much better than conventional in vitro assays. Co-culture of endothelial cells and fibroblasts or pericytes self-assemble their stable and perfusable microvasculatures within the three-dimensional microenvironment. Although in vitro tissue models do not completely mimic the complexity of in vivo conditions [17], these models might provide a unique system to investigate the efficacy, toxicity, and mode of action of therapeutic agents, such as nintedanib.

The objective of this study was to investigate how the anti-fibrotic drug nintedanib acts on vascular remodeling on an isolated 3D microvasculature network. Firstly, we established a 3D, stable and perfusable microvasculature network by co-culturing primary human lung fibroblasts and primary human umbilical vascular endothelial cells (HUVECs) within 3D fibrin gel network. Secondly, we explored the impact of nintedanib in this in vitro model of microvasculature architecture by investigating changes in vascular permeability, vessel density, fibroblast invasion, and endothelial-fibroblast interactions.

## Materials and methods

### Chip fabrication

The microfluidic device and fabrication process have been previously described in detail [12]. The chip used in our study is 100 µm in height, with five compartments, three chambers, and two microchannels separated by trapezoidal micropillars. The micropillars were spaced 100 µm apart, the diameter of the central circular chamber was 2 mm, and the adjacent microchannels and outermost chambers are 1 and 0.5 mm wide, respectively. To prepare the chip, a mixture of polydimethylsiloxane (PDMS; Sylgard) and curing agent was cast on a master mold fabricated by photolithography. After cutting and punching the access reservoirs, the PDMS pieces were treated with oxygen plasma and bonded to coverslips.

### Cell culture

Primary HUVECs (PCS-100-010; ATCC, USA) were cultured with endothelial growth medium 2 (EGM2; Lonza). Normal human lung fibroblasts (NL-FBs) were acquired from healthy lung tissue from patients undergoing tumor resection at the University Hospital, Bern, Switzerland, and cultured as mural cells with Ham’s F-12K (Kaighn’s) cell culture medium supplemented with 10% fetal bovine serum (FBS) and 1% penicillin–streptomycin. Details of the NL-FBs are available elsewhere [18]. Patients gave informed consent and primary cell culture was approved by the local ethical committee. For all experiments, HUVECs and NL-FBs were used between passage four and nine.

### Cell seeding and chip maintenance

Microfluidic chips were sterilized in an ozone chamber (CoolCLAVE) before seeding cells. HUVECs and NL-FBs were suspended in 2 U/ml bovine plasma thrombin (Sigma) in endothelial basal medium 2 (EBM2; Lonza) at final concentrations of 2 × 10^7^ and 1 × 10^7^ cells/ml, respectively. A solution of 10 mg/ml fibrinogen from bovine plasma (Sigma) in PBS (Gibco) was prepared. For co-culturing, HUVECs, NL-FBs, and fibrinogen solution were mixed at a ratio of 1:1:2 and immediately seeded into the central chamber. Next, a 1:1 mixture of NL-FBs and fibrinogen solution was immediately loaded in the outermost chambers. After 10 min, EGM2 was loaded in the microchannels and the reservoirs were filled. All chips were incubated at 37 °C with 5% CO_2_. Further details of chip co-seeding are available elsewhere [12]. A solution of 10 mM nintedanib (BIBF 1120) was prepared in 1 ml DMSO (S1010; Selleck Chemicals) and diluted for experimental use in EGM2. Treatment with nintedanib started on day 1, and both treated and untreated samples were cultured for 7 days. Medium (with or without nintedanib) was exchanged every 24 h. During medium exchange, a transient pressure difference was created to ensure flow of the new medium to cells in the central circular chamber.

### Immunostaining

The immunofluorescence staining protocol has been described previously [12]. Briefly, samples were incubated overnight with a primary antibody to the endothelial marker PECAM-1 (Santa Cruz) diluted 1:200 in 2% BSA in PBS. Samples were incubated with secondary antibodies conjugated with 488 Alexa Fluor (Molecular Probes), phalloidin (1:100; Invitrogen), and Hoechst (1:1000; Invitrogen) for 2 h at room temperature. All images were collected using a Zeiss LSM 710 confocal laser scanning microscope (Carl Zeiss Microscopy, LLC, Thornwood, NY).

### Live cell imaging

To observe the effect of nintedanib on endothelial-fibroblast interaction and fibroblast invasion, cell membrane labeling was carried out with the lipophilic cell tracker dyes PKH67 for HUVECs (green) and PKH26 for NL-FBs (red) before chip loading. Two different strategies were used for cell seeding and treatment. In all chips, NL-FBs in fibrin gel were seeded into the two outermost chambers, but some chips contained a mixture of HUVECs and NL-FBs in fibrin gel in the central chamber, whereas others had only HUVECs in fibrin gel. To study the effect of nintedanib on vasculogenesis, chips were treated with 50 nM nintedanib in EGM2 for 7 days. To evaluate the effect of nintedanib on angiogenesis, chips were treated with 50 nM nintedanib only on days 4–7, after formation of the premature microvasculature network. The chips were placed in an Omnitray, transferred to the Nikon BioStation CT, and imaged every 2 h. Chips remained inside the machine for 7 days, and medium was exchanged every 24 h.

### Vascularized area and diameter quantifications

In the central chamber, PECAM-1-positive regions were defined as vascularized areas. Z-stacks obtained from confocal microscopy were projected on a plane with maximum signal intensity using Fiji image analysis software [19]. Using the DiameterJ Segment plugin in Fiji [20], projected images were segmented using eight different thresholding techniques, and the most resembling segmentation to the original image was analyzed. The segmented image was analyzed using the DiameterJ 1-018 plugin [20] to quantify diameter distribution, number of intersections, and minimal and maximal diameters. Vascularized area fraction was defined as the area covered by vessels divided by the whole area of the central chamber and measured using the “Measure” option in Fiji.

### Microvessel permeability measurement

To assess the impact of nintedanib on microvessel wall integrity, vascular permeability was calculated as described previously [21]. Briefly, at the end of day 7, medium was removed from all reservoirs and 70 kDa rhodamine isothiocyanate (RITC)-conjugated dextran (Sigma) was added to one of the reservoirs at a concentration of 1 mg/ml in PBS. Starting immediately before RITC-dextran loading, images were acquired every 10 s using a Leica DMI 4000 microscope. The microvascular permeability coefficient was calculated based on the intensity of the fluorescence dye across the vascular wall over time using Fiji software. Five different regions per chip were quantified.

### Colocalization measurement

Colocalized area, defined as areas of overlapping HUVECs and NL-FBs in the central chamber, was calculated to quantify the effect of nintedanib on fibroblast localization around endothelial vessels. Images from the red (NL-FB) and green (HUVEC) channels were obtained from the Nikon BioStation CT on day 7, merged, and quantified using the Coloc plugin in Fiji [19] for colocalization analysis via intensity measurement. An image of overlapping areas was produced and then analyzed to obtain the area fraction measurement. At least five different areas per chip were quantified to calculate this percentage.

### Cellular health assessment

The proliferation rate of mono- and co-cultured HUVECs and NL-FBs was determined using the Cell Viability Imaging Kit (Blue/Green; ReadyProbes) according to the manufacturer’s protocol. Cells were resuspended in EGM2 and seeded into 96-well plates at densities of 2500 cells per well (HUVECs), 600 cells per well (NL-FBs), or 1600 cells per well (co-culture of HUVECs and NL-FBs). Nintedanib was diluted in EGM2 and added to cells at final concentrations of 10, 50, and 100 nM. Plates were seeded in duplicate to perform the assay twice, on days 4 and 7. A cocktail consisting of two drops each of stable NucBlue® Live (Hoechst 33342) and NucGreen® Dead reagents in 1 ml media was prepared, and 80 µl was added to each well. Samples were incubated for 15 min at room temperature then imaged using a Leica DMI 4000 microscope. At least two regions of each well were imaged. After binarizing the image and using “Particle Analysis” in Fiji, the numbers of live and dead cells were obtained. In addition to the proliferation assessment, the alamarBlue assay was used to evaluate cellular health after treatment with nintedanib. A 96-well plate was prepared as described above and treated with various concentrations of nintedanib. On day 7, the alamarBlue assay was performed according to the manufacturer’s protocol. After 2 h of incubation with alamarBlue, the absorbance was measured using a plate reader (TECAN). Absorbance values were corrected for background absorbance. Viability percentages reflect the ratio of the each absorbance value to average of control (0 nM) for each cell-culturing condition.

### Statistics

All data represent mean ± standard deviation of number of cases. Statistical variations between groups were analyzed using an unpaired *t* test, and *p* values less than 0.05 were considered statistically significant. Statistical analysis was performed using GraphPad Prism 6 software.

## Results

### Establishment of in vitro perfusable microvasculature network

**Fig. 1:**
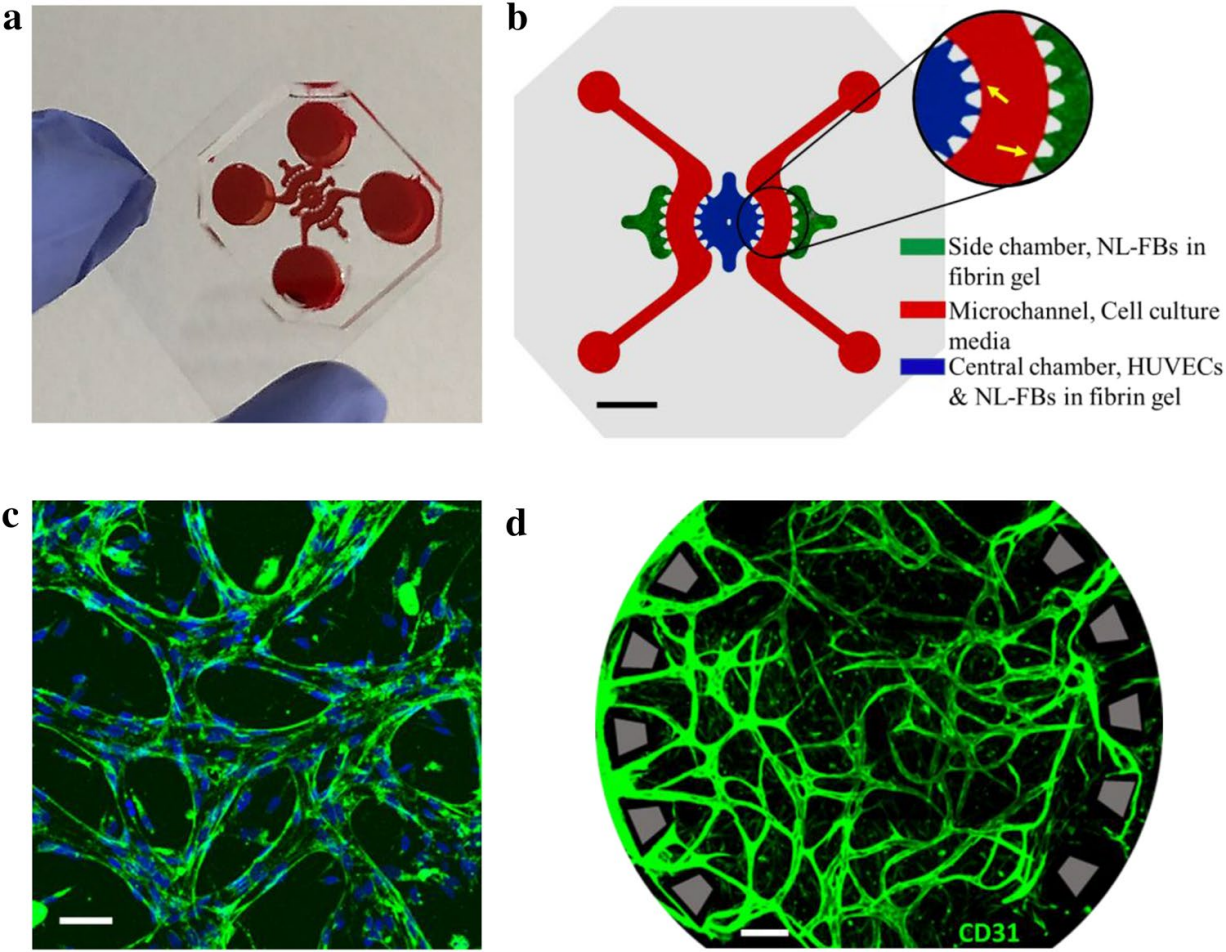
Microfluidic chip specifications and establishment of in vitro perfusable microvasculature network. **a** Microfluidic chip fabricated by PDMS soft lithography and bonded to coverslip, filled with red silicon dye for better visualization of the compartments. **b** Layout of the chip: NL-FBs in fibrin gel in the two outermost chambers (green), co-culture of HUVECs and NL-FBs in fibrin gel in the circular central chamber (blue), and cell culture media (red) in two symmetric microchannels. Close-up view shows the pillars of different compartments, indicated by yellow arrows. Scale bar: 2 mm. **c** Representative image showing the formation of the interendothelial junctions through the expression of PECAM-1 between adjacent HUVECs. Blue: DAPI, green: PECAM-1. Scale bar: 100 µm. **d** Representative image of the microvasculature network immunostained for endothelial cell marker PECAM-1 (green). This image from the central chamber was taken after 7 days of co-culturing. Scale bar: 200 µm

Flow channels were connected to the central chamber and side chambers via openings between trapezoidal pillars to supply nutrients and growth factors to cells located in these compartments (Fig. 1a, b). After 7 days of co-culturing HUVECs and NL-FBs (cell density ratio, 2:1), three-dimensional perfusable microvessels had formed in the central chamber. Immunostaining for PECAM-1 showed the existence of adherent junctions between endothelial cells that formed a microvasculature network (Fig. 1c, d).

Interconnectivity and perfusability are two key features of a functional vasculature model. To demonstrate the interconnectivity and perfusability of the vasculature network in our system, 70 kDa RITC-dextran was loaded in one of the reservoirs (Supplementary Material Video S1). Fluorescent dye flowed into the microvasculature network through the openings between the trapezoidal pillars. The calculated permeability coefficient was (1.5 ± 0.32) × 10^−6^ cm/s, which is similar to values measured for mammalian venules and other in vitro vasculature models [12, 22].

### Effect of nintedanib on cell proliferation and viability

**Fig. 2:**
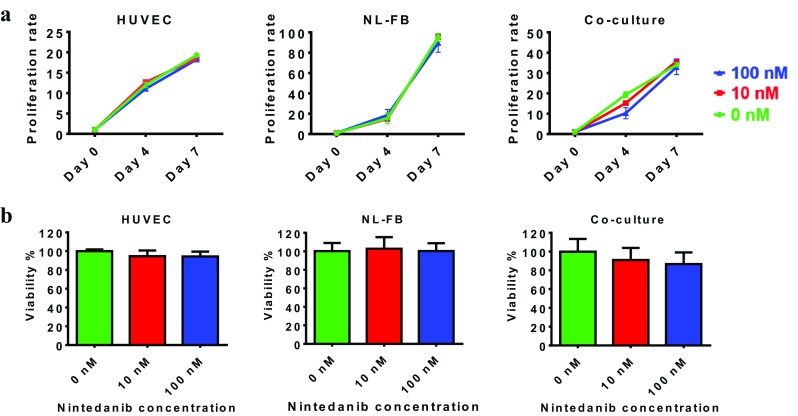
Cells maintain proliferation and viability when treated with nintedanib. **a** Proliferation rate of HUVECs, NL-FBs, and co-culture. Cells were imaged and counted after live/dead staining, and the proliferation rate was determined by calculating the ratio of live cells on each day to the number of cells originally seeded on day 0. Each condition was assessed in duplicate, with at least four sample images used for counting. **b** Viability of HUVECs, NL-FBs, and co-culture. The assay was performed in three independent experiments with duplicate wells

We performed live/dead staining of cells after 4 and 7 days of treatment with nintedanib. Nintedanib did not have an anti-proliferative effect on HUVECs, NL-FBs, or their co-culture in concentrations up to 100 nM (Fig. 2a). To assess the cytotoxicity of nintedanib, we utilized alamarBlue, a colorimetric assay in which absorbance is related to the number of living cells and depends on metabolic activity [23]. Nintedanib did not show cytotoxic effects and cells maintained viability after 7 days of treatment with nintedanib at concentrations up to 100 nM (Fig. 2b). The proliferation and viability of monocultures and co-cultures showed that 100 nM nintedanib was not cytotoxic in that context.

### Nintedanib increases microvascular permeability

**Fig. 3:**
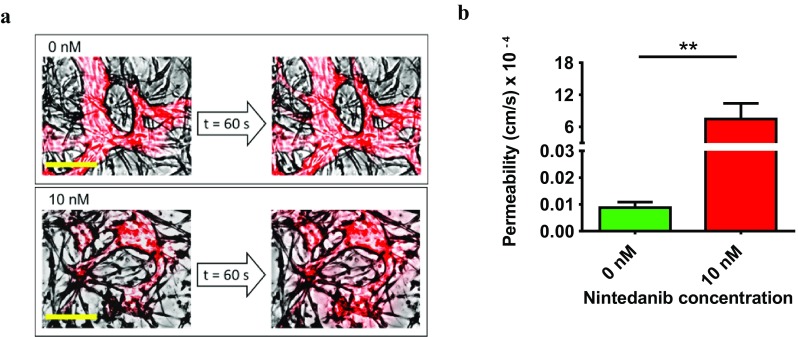
Nintedanib affected microvascular barrier integrity and permeability. **a** 70 kDa RITC-dextran was added to one of the reservoirs and filled the microvasculature in the central chamber. Images represent a segment of the central chamber and are overlays of the red channel and bright field. Time interval between the two images in each case is 1 min. In the 10 nM-treated chip, fluorescent dye leaked from the vasculature into the fibrin gel. The robust integrity of untreated microvessel walls kept the dye inside the vasculature and prevented any obvious leakage into the fibrin gel. Scale bar: 150 µm. **b** Time lapse images were recorded, and the vascular permeability coefficient was calculated from measurements of the fluorescence intensity across vascular walls over time. At least five areas per chip were analyzed. Treatment started on day 1. *n* = 6 and *p* < 0.0056

The particular design of our microfluidic chip enabled self-assembly of a perfusable microvasculature network in the central chamber, allowing us to investigate the action of nintedanib on microvascular barrier integrity and permeability. Untreated chips and chips treated with 10 nM nintedanib were perfused with 70 kDa RITC-dextran, and the fluorescent dye filled the microvasculature structures in the central chamber. In the presence of 10 nM nintedanib, dye leaked into the interstitial space, whereas no leakage from the microvasculature wall was observed in untreated networks (Fig. 3a). The calculated permeability coefficients were (1.5 ± 0.32) × 10^−6^ cm/s for untreated chips and (1.04 ± 0.36) × 10^−3^ cm/s for chips treated with 10 nM nintedanib. Thus, nintedanib treatment significantly (*p* < 0.0056) increased microvascular permeability (Fig. 3b). Permeability coefficients were calculated for untreated and 10 nM nintedanib-treated chips because, at higher concentrations of nintedanib, formation of perfusable microvasculature architecture was inhibited. In the untreated and 10 nM nintedanib settings, microvasculatures were more streamlined and open to the side microchannels through adjusted trapezoidal openings, whereas networks treated with 30 and 100 nM nintedanib had more tortuous microvessels that were largely closed to the access pillars (Supplementary Material Fig. S1).

### Nintedanib decreases microvasculature density

**Fig. 4:**
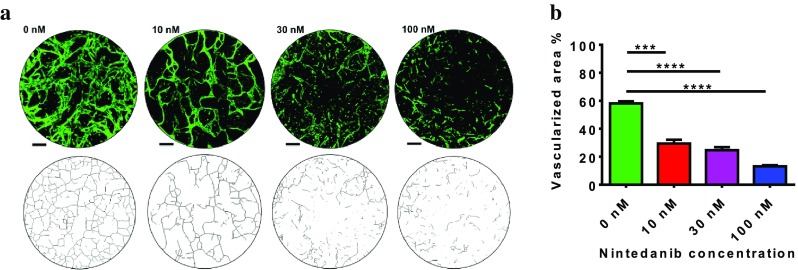
Nintedanib reduces density of in vitro microvessel architecture. **a** Top row: representative images of vascular morphologies of untreated chips and nintedanib-treated chips. Cells were immunostained for PECAM-1 (green). Images are projections of z-stacks acquired by confocal microscopy. Scale bar: 150 µm. Bottom row: axial thinning of microvasculature networks for better visualization of vascular intersections. Nintedanib decreases the number of vascular intersections and the amount of vascular sprouting. **b** Vascularized area was defined as the ratio of PECAM-1-positive areas to the whole area of the central chamber. Calculated vascularized areas were as follows: 58.13 ± 1.45% (untreated chip), 29.4 ± 2.71% (10 nM nintedanib), 24.67 ± 1.2% (30 nM nintedanib), and 13.1 ± 0.8% (100 nM nintedanib). Three regions were examined for each condition *p* < 0.0007

The PECAM-1 protein, encoded by the *PECAM1* gene, makes up a large portion of endothelial cell intercellular junctions and is widely recognized as an endothelial cell marker [24]. To quantify the effects of nintedanib treatment on microvasculature networks, we defined PECAM-1-positive regions in the central chamber as vascularized areas and analyzed the amount of vascularized area as well as network features and morphology. Representative images show that, in addition to reducing vessel density, nintedanib treatment altered microvessel morphology (Fig. 4a). For clearer visualization of these differences, we performed axial thinning of the networks using the DiameterJ 1-018 plugin in Fiji. Nintedanib treatment clearly decreased the quantity of vascular intersections and sprouting (Fig. 4a). Increasing concentrations of nintedanib led to a decrease in the total amount of vascularized area in the central chamber, indicating that nintedanib reduces microvasculature density in vitro (Fig. 4a). In untreated chips, approximately 58% of the central chamber was covered by the microvasculature network, whereas 100 nM nintedanib treatment reduced microvasculature coverage to approximately 13% (Fig. 4b).

### Nintedanib shrinks microvessel diameter

**Fig. 5:**
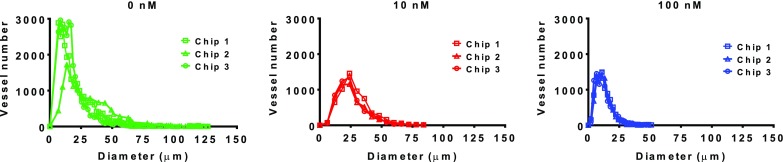
Diameter distribution of microvasculature network. Frequency of individual diameters in the vasculature network in untreated (0 nM) and nintedanib-treated chips (10 nM and 100 nM). Segmented microscopy images were analyzed by the DiameterJ 1-018 plugin. Nintedanib reduced the diameter of vessels and the frequency of vessels larger than 25 µm. *n* = 3 per condition. Treatment with nintedanib started on day 1

To assess the effect of nintedanib on microvasculature architecture, we examined the diameters of individual microvessels and obtained the diameter distribution of the network. Nintedanib had a thinning effect on microvessel diameter (Fig. 5, Fig. S3). In addition to shrinking vessel diameter, nintedanib dramatically decreased the number of vessels in the central chamber (Fig. S2).

### Nintedanib inhibits fibroblast organization around endothelial vessels

**Fig. 6:**
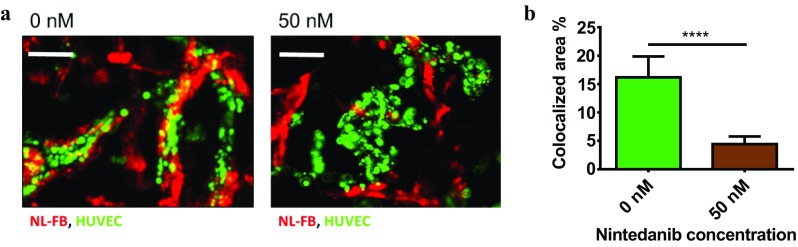
Fibroblasts organization in the microvessels. **a** Live cells were imaged, and segments of the central chambers on day 7 are shown. HUVECs are shown in green, and NL-FBs are shown in red. In untreated chips, NL-FBs appeared to wrap around the endothelial vessels, whereas treatment with 50 nM nintedanib caused NL-FBs to localize away from endothelial lumens. Nintedanib treatment started on day 1. Scale bar: 200 µm. **b** Colocalized areas were defined as regions where fibroblasts overlapped endothelial cells. Nintedanib decreased fibroblast coverage around endothelial cells and significantly decreased the amount of colocalized area

To examine the effect of nintedanib on endothelial–fibroblast interactions, we used membrane labeling to track live cells. We observed that nintedanib treatment affected the association of fibroblasts with endothelial vessels. In untreated chips, NL-FBs spread around microvascular segments and aligned with the microvessel, and NL-FBs clearly influenced vessel orientation. At the end of day 7, untreated vasculatures had developed into streamlined endothelial vessels externally wrapped by fibroblasts (Fig. 6a). Treatment with 50 nM nintedanib inhibited the alignment of NL-FBs around endothelial microvessels; NL-FBs located in the vicinity of vessels did not spread around the vessels. After 7 days of 50 nM treatment, microvasculatures were tortuous and lacked an exterior sheath of fibroblasts (Fig. 6a). Quantification of the colocalization of HUVECs and NL-FBs revealed that nintedanib significantly inhibited fibroblast organization around endothelial vessels (Fig. 6b).

### Nintedanib prevents fibroblast invasion

**Fig. 7:**
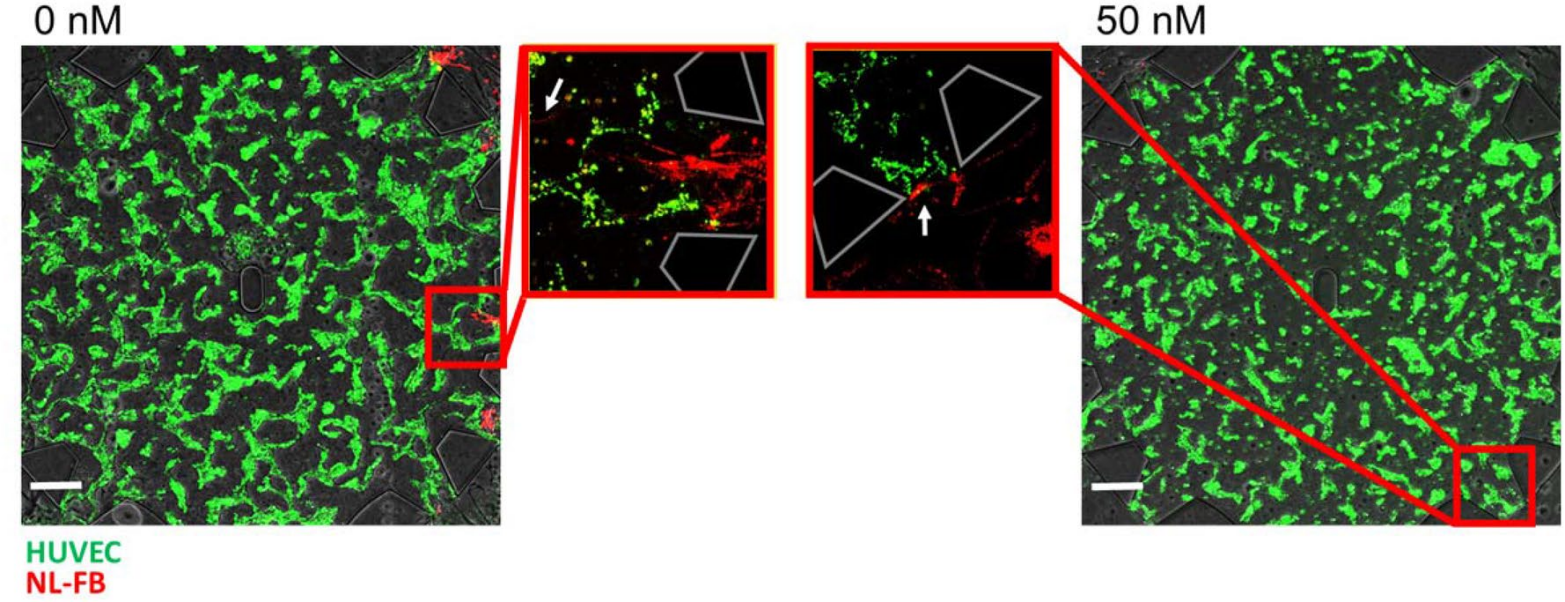
Fibroblasts microvasculature invasion. Representative overlay images of central chambers on day 7. Trapezoidal structures indicate the pillars around the central chamber. White arrows show the location of the fibroblasts. In untreated chips, NL-FBs (red) migrated across the microchannel and through the entrances to the central chamber filled with HUVECs (green), but treatment with 50 nM nintedanib inhibited migration and invasion through the fibrin gel. Scale bars: 200 µm

To achieve a clear understanding of nintedanib influence on fibroblast migration, we wished to observe the movement of fibroblasts from the side chambers toward the endothelial cells in the central chamber. Cells were seeded separately: green-labeled HUVECs in the central chamber and red-labeled NL-FBs in the side chambers. In untreated conditions, fibroblasts migrated within the flow microchannels toward the endothelial cells and invaded the central chamber by migration through the fibrin gel. After treatment with 50 nM nintedanib, NL-FBs reached the perimeter of the central chamber via the microchannels, but remained in the entrances and did not move into the central chamber (Fig. 7, Fig. S4). Additionally, 50 nM nintedanib treatment caused fewer fibroblasts to localize near the central chamber (data not shown). In untreated chips, fibroblast invasion of the central chamber occurred through a majority of the openings, whereas in 50 nM nintedanib-treated chips, fibroblasts did not migrate through any of the openings.

### Nintedanib breaks vessel–vessel connection in pre-existing microvasculature network

**Fig. 8:**
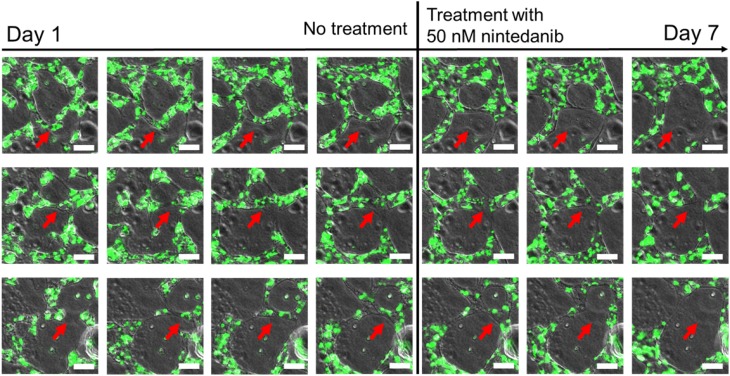
Remodeling of pre-existing microvasculature network. Images represent vascular remodeling in three different segments of the central chamber on days 1–7. Nintedanib treatment (50 nM) started on day 4. HUVECs are shown in green. Red arrows indicate the regions where vessel–vessel connections progressively disappeared. Scale bar: 60 µm

To address the effect of nintedanib on remodeling of a preformed microvasculature network, we modified our treatment protocol such that cells were seeded on chips as for other experiments, but 50 nM nintedanib treatment started on day 4, after the premature vasculature network formed, rather than on day 1. Sprouting, vascular formation, and lumen formation of endothelial cell clusters with neighboring cells took place from day 1 to day 4 (Fig. 8). After addition of 50 nM nintedanib on day 4, existing vessel–vessel connections gradually weakened and finally disappeared (Fig. 8). In addition to its anti-vasculogenic effect, nintedanib remodeled the pre-existing microvasculature network by shrinking intervascular connections and inhibiting vascular sprouting (Supplementary Material Video S2).

## Discussion

Advanced in vitro microfluidic models enable visualization of biological dynamics, elimination of the interspecies variations, and reduction of the amount of cells and reagents to be used. In addition, these models offer a controllable investigation of vascularization to develop on-chip models that can be used in the future for optimization and development of drug treatments on personalized vasculature networks [25]. In this study, for the first time, fibroblasts acquired from healthy lung tissue of patients were utilized to establish a 3D, stable and perfusable microvasculature network in vitro. After establishment of this network, we investigated the effects of the triple tyrosine kinase inhibitor nintedanib on in vitro human microvasculature system. We demonstrated the impact of nintedanib on vascular permeability, vessel density, vascular morphology, endothelial–fibroblast interaction, and fibroblast invasion in vitro. Using our microvasculature-on-chip model, we showed that treatment with nintedanib significantly increased vascular permeability, decreased vessel density by both reducing number of vessels and shrinking the vessels themselves, inhibited fibroblast recruitment to endothelial vasculatures, constrained fibroblast invasion toward the vasculature region, and weakened vessel–vessel connections of existing microvasculatures.

Before testing nintedanib in our model, we assessed its cytotoxicity on monoculture and co-culture of HUVECs and NL-FBs in concentrations up to 100 nM. Alamar blue and live-dead staining assays revealed that all cell cultures maintain their viability and proliferation when exposed to up to 100 nM treatment with nintedanib. This result stands in contrast to a previous report, where treatment of either VEGF- or bFGF-stimulated HUVECs with nintedanib resulted in inhibition of cell proliferation and apoptosis with *EC*_50_ values of less than 10 and 290 nM, respectively [6]. However, in the present study, HUVECs were stimulated with both VEGF and FGF (which were present in the fully supplemented EGM-2 media), so they were expected to be more durable against the toxic effect of nintedanib. Previous studies showed no evidence of acute toxicity on human lung fibroblasts at nintedanib concentrations up to 1 µM [26]. It has also been shown that nintedanib inhibits proliferation of lung fibroblasts significantly only at concentrations greater than 500 nM [26].

Interestingly, although nintedanib did not have adverse effect on cellular viability and proliferation, when administered on-chip during microvessel formation, it affected the in vitro microvasculature at concentrations as low as 10 nM. For direct comparison, the concentrations we selected for nintedanib to be administered on-chip is approximately between 0.7 and 1.4 times of its concentration in human plasma [27].

Furthermore, nintedanib-treated in vitro vessels showed a significant increase in vascular leakage across the vessel wall. On-chip administration of nintedanib increased the permeability of in vitro microvessels by three orders of magnitude. Nintedanib also prevented fibroblasts from elongating and ensheathing endothelial vessel segments, possibly by interfering with paracrine PDGF signaling between endothelial cells and fibroblasts. PDGF is responsible for the chemoattraction and proliferation of pericytes and fibroblasts in the vessel walls [5]. In IPF, in addition to fibroblasts, PDGF is found in endothelial cells and epithelial cells which suggests contribution of PDGF in vascular remodeling [28]. Inhibiting PDGF/PDGFR signaling cascade interferes chemoattraction and migration of fibroblasts and results in formation of unstable network of the microvasculatures. The presence of fibroblasts is known to directly affect the permeability and morphology of microvasculature in vitro; perivascular cells wrap around vessels, stabilize the intercellular junctions, increase basement membrane deposition, and thus decrease the permeability of the microvasculatures [12, 13, 29–31]. Therefore, the high leakage could be explained by lack of perivascular coverage and junctional stability via PDGF signaling.

Ackermann et al. reported that nintedanib increased the intervessel distance in vivo by a factor of 1.7 and decreased vessel diameter from 5.1 ± 1.38 to 3.71 ± 1.0 µm, indicating a significant reduction in vessel density [10]. These results are consistent with our data showing that treatment with nintedanib considerably decreases the vascular density and diameter in vitro. In addition to modulating several signaling pathways and controlling endothelial cells during angiogenesis, VEGF is the master regulator of vessel formation and sprouting [32]. Although the precise mechanism of VEGF in angiogenesis is not fully elucidated, variety of biologic responses have been reported for VEGF–VEGFR binding: inhibiting endothelial cells apoptosis, regulating migration and proliferation of these cells, increasing vascular permeability and maintaining vascular integrity [11, 33–36]. To regulate sprouting, the spatial distribution of VEGF loosens cell–cell adherence, directs tip cell migration and determines stalk cells proliferation [37]. Inhibition of VEGF/VEGFR signaling could have harmful effect on vascular integrity by promoting endothelial cells apoptosis [11]. Additionally, VEGF blocking down regulates vascular sprouting and decreases capillary branching [28]. Interestingly, blockade of VEGF with specific antibodies was shown to decrease the number of capillary-like tubes by up to 70%, further corroborating our finding [31]. FGF is heparin binding growth factor that induces endothelial cells proliferation and migration [38]. FGF is also mediator of preliminary step in angiogenesis by contributing in ECM degradation through plasminogen activator expression [39]. Inhibition of FGF/FGFR signaling could constrain endothelial cells migration, cause prevention of ECM degradation and show similar effect on sprouting as VEGF/VEGFR blocking.

The invasion of human lung fibroblasts was recently studied using the Matrigel invasion assay [26]. The presence of nintedanib in the cell culture medium reduced the invasion capability of lung fibroblasts [26]. The specific design of our microvasculature platform enabled examination of the invasion of NL-FBs from fibrin gel into EGM2 and also into fibrin gel with incorporated HUVECs. The low density of lung fibroblasts at the entrances of the central chamber suggests that NL-FBs are constrained from invading into a medium in the presence of nintedanib. Several in vivo studies about the effect of nintedanib on vascular density in lung cancer and fibrosis models revealed that nintedanib acts on pre-existing microvessel networks [10, 40]. Similarly, we found that in addition to inhibiting vasculogenesis, nintedanib remodels pre-existing microvessels by shrinking and rupturing intravascular connections.

We sought to explore the anti-angiogenic and anti-vasculogenic effects of nintedanib with the goal of evaluating its use in treatment of IPF, but our approach has two caveats. We utilized HUVECs in our in vitro microvasculature model, but HUVECs differ from lung vascular endothelial cells in terms of gene expression and morphology [10, 41]. We also utilized normal lung fibroblasts, which have different levels of PDGFR and FGFR expression than IPF fibroblasts [7]. Ideally, the microvasculature architecture in the in vitro model would mimic IPF pathology, which may be possible if fibroblasts from IPF patients were seeded into the chip with endothelial cells. Such an in vitro diseased microvasculature model would be very useful for drug discovery and testing.

Advanced in vitro microvasculature models open new prospects to study the mode of action of treatment agents such as nintedanib. These experimental platforms can address fundamental questions of drug effects on angiogenesis and vasculogenesis and can be used for developing and optimizing drug treatments in personalized vasculature models in the future. In summary, our work revealed that nintedanib treatment at clinically relevant concentrations did not affect cell viability but did significantly affect neovasculogenesis by loosening vascular barrier integrity, increasing permeability, shrinking microvessels, decreasing vascular density, and preventing fibroblast organization around endothelial vasculatures. The effect of nintedanib on microvasculature architecture has previously been studied in vivo [10], and our in vitro 3D model of human microvasculature represents an exciting approach because it permits to study of the effect of nintedanib on isolated human microvessels and resolve cell–cell interactions in greater detail than in vivo studies.

## Electronic supplementary material

Below is the link to the electronic supplementary material.

Supplementary material 1 (AVI 1510 KB)

Supplementary material 2 (AVI 2682 KB)

Supplementary material 3 (DOCX 1584 KB)
