# Supplementary material for: Human microvasculature-on-a chip: anti-neovasculogenic effect of nintedanib in vitro
Source: Angiogenesis. 2018 Jul 2;21(4):861–71. doi: 10.1007/s10456-018-9631-8 (PMC6208892; doi:10.1007/s10456-018-9631-8)
Supplement: Supplementary file 3 — Supplementary material 3 (DOCX 1584 KB) [file 10456_2018_9631_MOESM3_ESM.docx]

Supplementary Figures

| a | 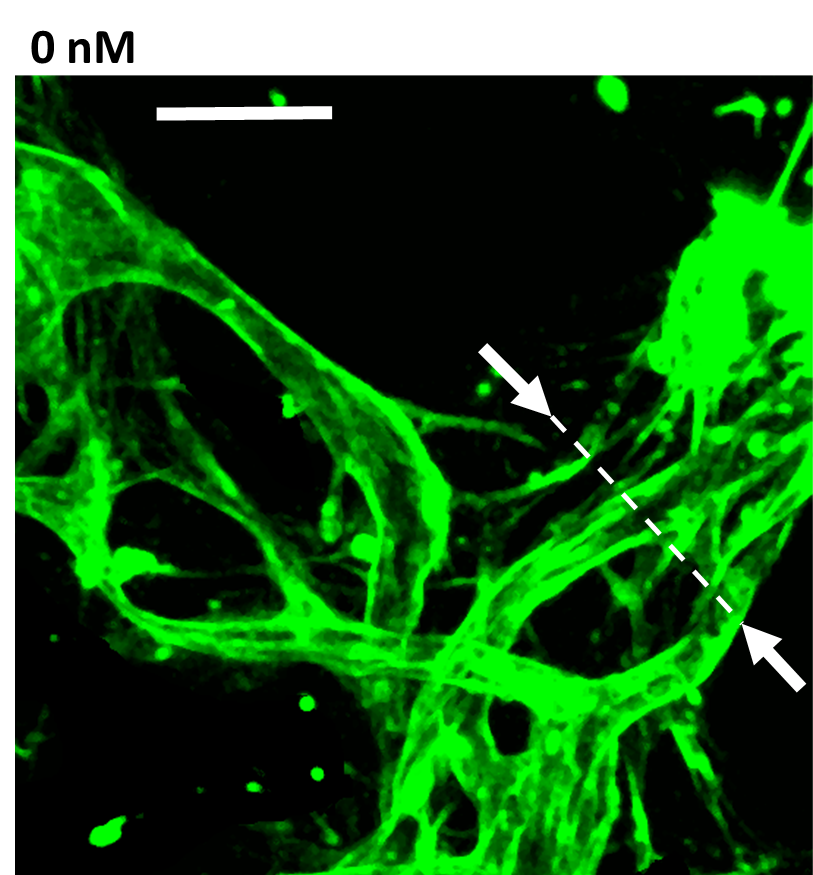 | b | **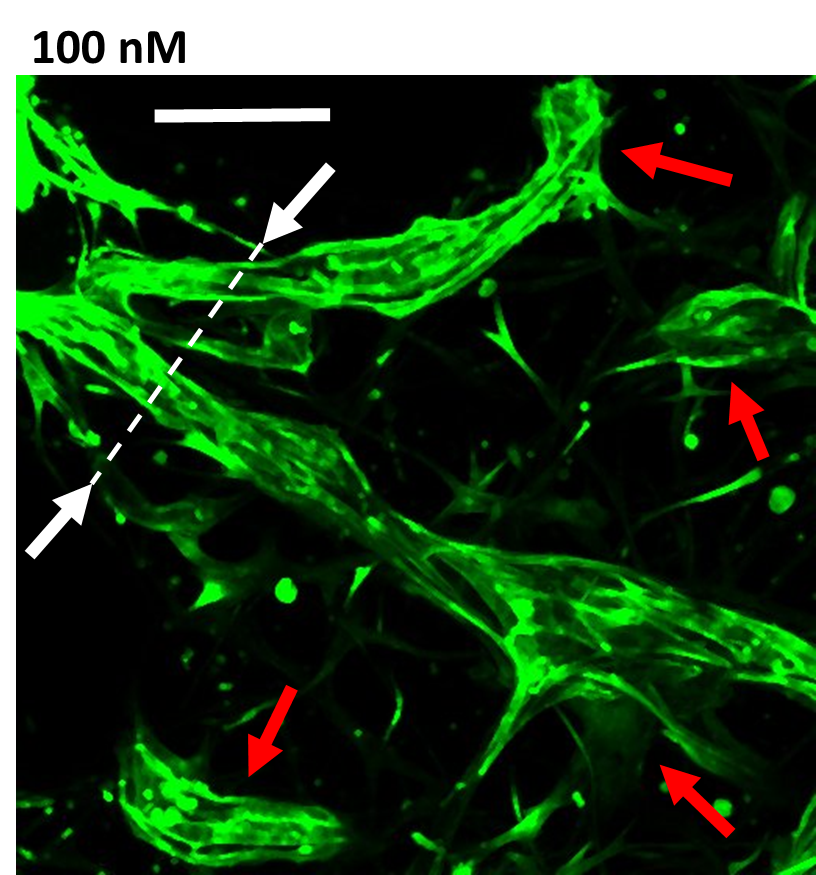** |
| --- | --- | --- | --- |
| **Fig. S1** Morphology of the microvasculature  (a) In the absence of nintedanib, microvasculatures were more streamlined and open to the side microchannels through adjusted trapezoidal openings. The white arrows and dashed line indicate the location of the opening pillar from the microchannel to the central chamber. Cells were immunostained for PECAM-1 (green). Scale bar: 100 µm. (b) Treatment with 100 nM nintedanib caused microvessels to have a more tortuous morphology, and they were largely closed to the access pillars. The white arrows and dashed line indicate the location of the opening pillar from the microchannel to the central chamber. Red arrows indicate tortuous microvessels in the central chamber. Cells were immunostained for PECAM-1 (green). Scale bar: 100 µm. | | | |

**Fig. S2** Distribution of vessel diameter range

Vasculature diameters of untreated and nintedanib-treated chips were categorized in three different ranges: 0-15µm (small), 16-50 µm (medium) and 50+ µm (large). In all vasculature categories, nintedanib decreased number of microvessels. Number of vessels in each category was quantified and then compared between treated and untreated cases.

**Fig. S3** Maximum vessel diameter in untreated and nintedanib-treated microvasculature networks. Nintedanib has significant thinning effect on microvasculatures.

**Fig. S4** Invasion of NL-FB into the central chamber in untreated and 50 nM nintedanib-treated chips.

In 50 nM nintedanib-treated chips, NL-FBs did not migrate into the central chamber. Quantities of y-axis reflect the distance of migrated NL-FB into the central chamber from periphery of the central chamber. NL-FB invasion from seven entrances per chip has been determined.
